# A prospective multicenter study on the evaluation of antimicrobial resistance and molecular epidemiology of multidrug-resistant *Acinetobacter baumannii* infections in intensive care units with clinical and environmental features

**DOI:** 10.1186/s12941-019-0319-8

**Published:** 2019-07-02

**Authors:** Baris Boral, Özlem Unaldi, Alper Ergin, Riza Durmaz, Özgen Köseoğlu Eser, the Acinetobacter Study Group, Pinar Zarakolu, Gülden Ersöz, Ali Kaya, Demet Haciseyitoglu, Öznur Ak, Serap Gencer, Fatma Mutlu Sarigüzel, İlhami Çelik, Muhammet Hamdullah Uyanik, Kemalettin Özden, Ziya Cibali Acikgöz, Rahmet Guner, Alper Akcali, Alper Sener, Ali Adiloglu, Cemal Bulut, Meltem Yalinay, Murat Dizbay, Söhret Aydemir, Oguz Resat Sipahi

**Affiliations:** 1 Department of Medical Microbiology, Hacettepe University Faculty of Medicine, Ankara, Turkey; 2 Department of Microbiology Reference Laboratories, National Molecular Microbiology Reference Laboratory, Ankara, Turkey; 3 Hacettepe University School of Health Services, Ankara, Turkey; 4 Department of Medical Microbiology, Ankara Yildirim Beyazit University Faculty of Medicine, Ankara, Turkey

**Keywords:** *Acinetobacter baumannii*, Risk factors, Antimicrobial resistance genes, Polymerase chain reaction, Pulsed field gel electrophoresis, Clonal relatedness

## Abstract

**Background:**

Multidrug-resistant (MDR) *Acinetobacter baumannii* infections are considered as emerging nosocomial infections particularly in patients hospitalized in intensive care units (ICUs). Therefore, reliable detection of MDR strains is crucial for management of treatment but also for epidemiological data collections. The purpose of this study was to compare antimicrobial resistance and the clonal distribution of MDR clinical and environmental *A*. *baumannii* isolates obtained from the ICUs of 10 different hospitals from five geographical regions of Turkey in the context of the demographic and clinical characteristics of the patients.

**Methods:**

A multicenter-prospective study was conducted in 10 medical centers of Turkey over a 6 month period. A total of 164 clinical and 12 environmental MDR *A. baumannii* isolates were included in the study. Antimicrobial susceptibility testing was performed for amikacin (AN), ampicillin–sulbactam (SAM), ceftazidime (CAZ), ciprofloxacin (CIP), imipenem (IMP) and colistin (COL) by microdilution method and by antibiotic gradient test for tigecycline (TIG). Pulsed-field gel electrophoresis (PFGE) was performed to determine the clonal relationship between the isolates. The detection of the resistance genes, *bla*_OXA-23_, *bla*_OXA-24_, *bla*_OXA-51,_ *bla*_OXA-58,_ *bla*_IMP,_ *bla*_NDM_, *bla*_KPC_, *bla*_OXA-48_ and *bla*_PER-1_ was carried out using the PCR method.

**Results:**

The mortality rate of the 164 patients was 58.5%. The risk factors for mortality included diabetes mellitus, liv1er failure, the use of chemotherapy and previous use of quinolones. Antimicrobial resistance rates for AN, SAM, CAZ, CIP, IMP, COL and TIG were 91.8%, 99.4%, 99.4%, 100%, 99.4%, 1.2% and 1.7% respectively. Colistin showed the highest susceptibility rate. Four isolates did not grow on the culture and were excluded from the analyses. Of 172 isolates, 166 (96.5%) carried *bla*_OXA-23_, 5 (2.9%) *bla*_OXA-58_ and one isolate (0.6%) was positive for both genes. The frequency of *bla*_PER-1_ was found to be 2.9%. None of the isolates had *bla*_IMP_, *bla*_KPC_, *bla*_NDM_ and *bla*_OXA-48_ genes. PFGE analysis showed 88 pulsotypes. Fifteen isolates were clonally unrelated. One hundred fifty-seven (91.2%) of the isolates were involved in 14 different clusters.

**Conclusions:**

Colistin is still the most effective antibiotic for *A*. *baumannii* infections. The gene *bla*_OXA-23_ has become the most prevalent carbapenemase in Turkey. The distribution of invasive *A*. *baumannii* isolates from different regions of Turkey is not diverse so, infection control measures at medical centers should be revised to decrease the MDR *A. baumannii* infections across the country. The results of this study are expected to provide an important baseline to assess the future prophylactic and therapeutic options.

## Introduction

Invasive infections due to *Acinetobacter baumannii* are among the leading nosocomial infections in patients hospitalized in intensive care unit (ICU) [1]. The increasing number of these infections and the emergence of multidrug-resistant (MDR) strains make *A*. *baumannii* a troublesome pathogen especially in a hospital environment [1]. *A. baumannii* accounts for almost 90% of all reported *Acinetobacter* infections including ventilator-associated pneumonia, bacteremia, meningitis, peritonitis, urinary tract infections and wound infections [2].

Hospital-acquired pneumonia represents the most common clinical manifestation of *A*. *baumannii* infections. *A. baumannii* is also a common cause of bloodstream infections in ICUs [3]. The most common sources of these infections are the patients having lower respiratory tract infections and intravascular devices [4]. Risk factors include immunosuppression, ventilator- associated respiratory failure, previous antibiotic therapy, colonization with *A*. *baumannii* and invasive procedures [5].

Initial effective treatment is key to increasing the survival rate in bacterial infectious diseases however providing such treatment is a major clinical challenge due to the high rate of antibiotic resistance. For instance, increasing carbapenem resistance in *A*. *baumannii* infections is a serious threat for causing a rise in healthcare costs and worsening clinical outcome. The main mechanism of carbapenem resistance in *A. baumannii* is hydrolysis of carbapenems with metallo-beta-lactamases and/or class D beta lactamases. Although metallo-beta-lactamases (VIM, IMP, SIM) have been reported especially in Asia and Western Europe, class D beta-lactamases (OXA-23, OXA-24 or -40, OXA-51, OXA-58, and OXA-143) have been identified in the United States, Latin America, Europe, Asia and many other parts of the world [6, 7]. Studies of the class D beta-lactamases show that especially the *bla*_OXA-23_ gene can be easily spread via transposons and conjugative plasmids [8]. The successful evolution of transposons bearing the *bla*_OXA-23_ resistance gene has an important role in spreading the resistant bacteria worldwide [8].

Various genotyping methods such as ribotyping, pulsed-field gel electrophoresis (PFGE), multilocus sequence typing (MLST), single locus sequence typing (SLST), and analysis of restriction fragment length polymorphism (RFLP) can be used to understand transmission dynamics of MDR *A*. *baumannii*. The PFGE technique is known as the gold standard for genotyping of *A*. *baumannii* strains [9].

Epidemiological studies on the clinical and molecular characteristics of *A*. *baumannii* strains might help to determine infection control strategies for different centers. The purpose of this study was to evaluate antimicrobial drug resistance mechanisms and the clonal distribution of clinical and environmental MDR *A*. *baumannii* isolates from ICUs in five geographical regions of Turkey and to compare these with the demographic characteristics of the patients.

## Methods

### Study design

A prospective multicenter study was conducted from 10 different medical centers located in five different geographical regions (Aegean, Central and Eastern Anatolia, Marmara, Mediterranean) of Turkey in 2012. All patients who were admitted to the ICUs of medical centers with the diagnosis of invasive *A*. *baumannii* infections were included in the study. The study was reviewed and approved by the Non-Interventional Clinical Research Ethics Committee of Hacettepe University, Ankara, Turkey.

### Patient characteristics and risk factors

The characteristics and risk factors of 164 patients with clinical and microbiological diagnosis of *A*. *baumannii* were analyzed. Data were collected prospectively in a standardized case report form. The following data were recorded in all centers: age, gender, occupation, immunosuppression, underlying diseases, previous hospitalization, duration of stay in the unit, invasive procedures or surgery within 30 days before the diagnosis, prior antibiotic treatment and outcome of treatment. Invasive procedures included central and urinary catheterization, mechanical ventilation, nasogastric tube placement, and orotracheal intubation.

### Sample collection and identification of the isolates

**Fig. 1:**
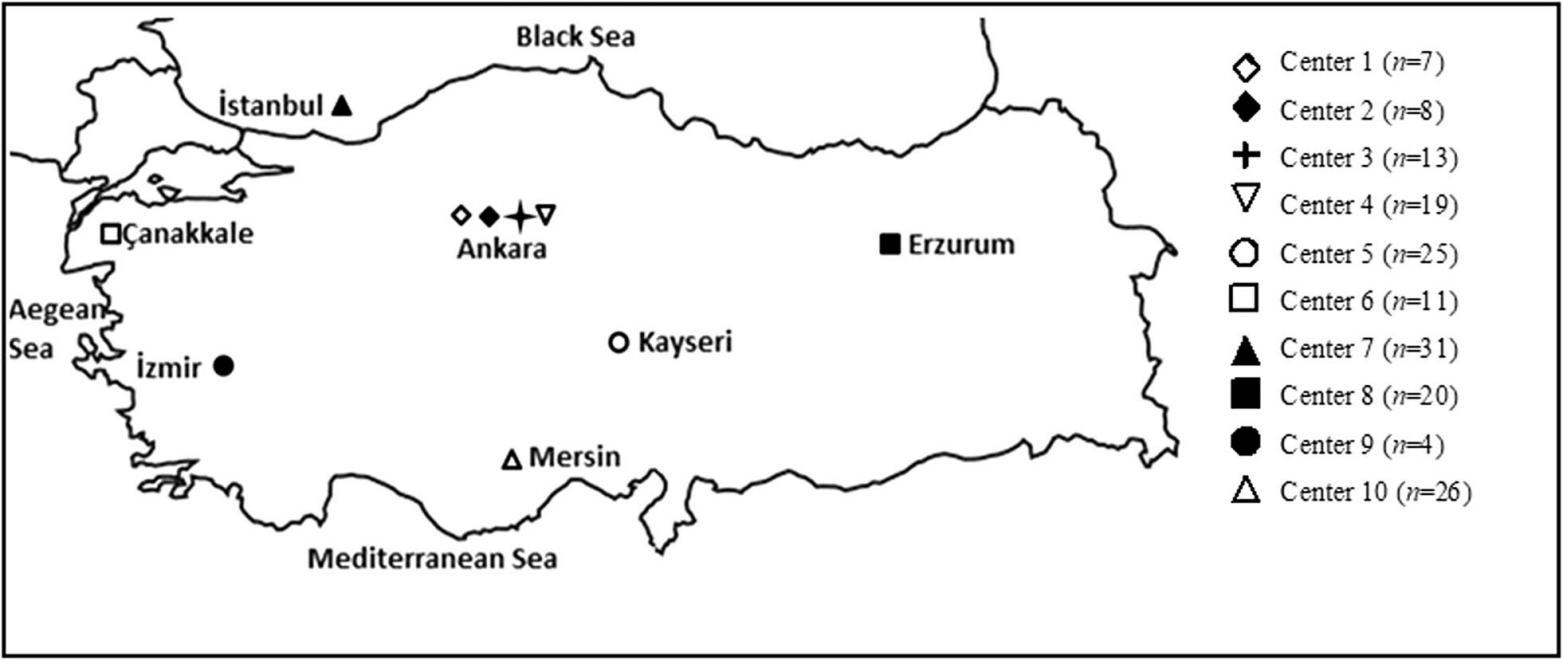
Distribution of centers collaborating in the study

A total of 164 clinical and 12 environmental *A*. *baumannii* isolates were included in the study. The participating centers were Hacettepe University Hospitals (HACET) (Center 1), Gazi University Hospital (GAZI) (Center 2), Ankara Education and Research Hospital (AH) (Center 3), Ankara Ataturk Education and Research Hospital (AEH) (Center 4), Kayseri Education and Research Hospital (KEAH) (Center 5), Canakkale Onsekiz Mart University Hospital (Center 6), Dr. Lutfu Kirdar Education and Research Hospital (Center 7), Ataturk University, Research Hospital (Center 8), Ege University Hospital (Center 9) and Mersin University Hospital (Center 10) (Fig. 1). All the patients had monomicrobial infection with MDR *A. baumannii*. Clinical isolates were obtained from patients hospitalized in medicine and surgery ICUs, with samples taken from blood (89%) and other sterile body sites [cerebrospinal fluid (CSF), bronchoalveolar lavage fluid (BAL), pleural fluid, tissue and catheter]. Environmental isolates were taken from table surfaces, sinks, taps and pumps from the ICUs in all centers.

All isolates were identified by automated systems [either the Vitek 2 system (bioMérieux, Marcy-l’Étoile, France) or Phoenix system (Becton–Dickinson, Franklin Lakes, NJ)] and they were confirmed at Hacettepe University Faculty of Medicine, Department of Medical Microbiology by conventional methods and also by molecular methods for confirming the existence of the *bla*_OXA-51_ gene. All the confirmed isolates were kept at − 80 °C during the data collection period.

### Antimicrobial susceptibility testing

Antimicrobial susceptibility tests were performed for six antimicrobials; ampicillin–sulbactam (SAM), amikacin (AN), ceftazidime (CAZ), ciprofloxacin (CIP), imipenem (IMP) and colistin (COL) by using the broth microdilution method according to Clinical Laboratory Standards Institute (CLSI) guidelines [10]. *Pseudomonas aeruginosa* ATCC 27853 strain was used as a reference control in all antibiotic susceptibility testings except for sulbactam–ampicillin. *Escherichia coli* ATCC 35218 was used as a reference control for this antibiotic. In addition, the minimal inhibitory concentration (MIC) value for tigecycline (TIG) was determined by antibiotic gradient strips, Etest (bioMérieux, France). Interpretation break points for TIG of ≤ 1 mg/L were considered as susceptible, and ≥ 4 mg/L as resistant and MIC values against tigecycline were evaluated using FDA-approved breakpoints provided in the package insert [11].

### Detection of antibiotic resistance genes

Two multiplex PCR protocols were carried out for the detection of resistance genes; one for *bla*_OXA-23_, *bla*_OXA-24_, *bla*_OXA-51,_ and *bla*_OXA-58_ and the other one for *bla*_IMP_, *bla*_NDM_, *bla*_KPC_, *bla*_OXA-48_ in all 172 isolates [12, 13]. The existence of the *bla*_PER-1_ gene was also determined by using a protocol which was described by Weldhagen et al. [14]. Each PCR reaction (25 μL) contained 12.5 μL of Taq PCR master mix (New England Biolabs, Beverly, MA), 5.5 μL sterile-RNase free water, 0.5 μL of each primer (100 μM, final concentration 2 μM) and 2 μL of DNA template. Amplified products were visualized on 2% agarose gel.

### Pulsed-field gel electrophoresis

PFGE of 172 isolates was implemented as previously described by Durmaz et al. [15]. Briefly, the genomic DNA was digested with *Apa*I (New England Biolabs, Beverly, MA). The plugs were embedded into 1% agarose gel wells. Subsequently, the separation of the DNA bands was carried out using the CHEF DR III system (Bio-Rad, Nazareth, Belgium) at 6 V/cm^2^ for 19 h and the pulse time was changed from 5 to 20 s. PFGE pattern analysis was carried out by using Bionumerics software version 4.0 (Applied Maths, Austin, TX) and with the unweighted-pair group method using average linkages. In order to analyze clusters Dice settings were used.

### Statistical analysis

Univariate conditional logistic regressions were used in the study group. Odds ratios were estimated by exponentiation of regression coefficients and their 95% confidence interval (CIs) reported. To adjust for confounding factors, variables with a *p* value below the 10% significance level in univariate analysis were entered in multiple conditional logistic regression models.

## Results

### Patient characteristics and risk factors

A total of 164 patients were included in the study. Of these patients, 88 (53.6%) were men and the age range was 15 days–78 years. The mean age of the patients was 60.1. Data from all 164 patients indicated that 52.4% of them were hospitalized in surgical ICUs, 30.5% in medical ICUs, 11% in burns units and 6.1% in emergency medicine service (Table 1). At least one underlying disease was identified in 95% of the patients. The most frequent diagnosis was ventilator-associated pneumonia (VAP) (26.6%) followed by malignancy (23%). The underlying diseases of patients were recorded as diabetus mellitus (DM), chronic kidney failure (CKF), chronic obstructive pulmonary disease (COPD), acute respiratory distress syndrome (ARDS), chronic liver failure (CLF) and febrile neutropenia (FEN). The risk factors for mortality contained DM [hazard ratio (HR): 2.74; *p *= 0.026], CLF (HR: 4.27; *p *= 0.047), chemotherapy (HR: 3.69; *p *= 0.03) and previous usage of fluoroquinolones (HR: 3.64; *p *= 0.01) (Table 2). According to available data, carbapenems (67/153) and third generation cephalosporins (51/153) were the most common antibiotics received by patients before the diagnosis of MDR *A*. *baumannii* infection.

**Table 1:** The demographic features of the patients and risk factors (n = 164)

	Number (*n*)	Frequency (*%*)
Gender		
Male	88	53.6
Female	76	46.3
Service		
Medical ICUs	50	30.5
Surgical ICUs	86	52.4
Burn ICUs	18	11
Emergency service	10	6.1
Immunosuppression		
Corticosteroid usage	18	11
Transplantation (solid organ, bone marrow)	–	–
Chemotherapy	15	9.1
Invasive procedures		
Central catheterisation	125	76.2
Urinary catheterisation	161	98.2
Mechanical ventilation	112	68.7
Nasogastric tube	86	52.4
Orotracheal intubation	74	45.4
Other	32	19.5
Mortality	96	58.5

*ICU* intensive care unit

**Table 2:** The risk factors of mortality among patients (n = 164)

	Mortality (*n *= 96)	Survival (*n *= 68)	Univariate analysis	
			Risk ratio (%95 CI)	*p* value
Demographic features				
Age			1.863 (1.251–2.775)	*0.001*
< 64	39	45		
> 65	57	23		
Gender			1.380 (0.0939–2.028)	0.093
Male	48	43		
Female	48	25		
Comorbidity				
Diabetes mellitus	23	7	2.746 (1.103–6.833)	*0.026*
Chronic obstructive pulmonary diseases	12	6	1.476 (0.525–4.149)	0.458
Congestive heart failure	12	8	1.071 (0.413–2.781)	0.887
Chronic liver failure	11	2	4.271 (0.915–19.932)	*0.047*
Corticosteroid usage	8	11	0.471 (0.179–1.242)	0.122
Neutropenia	4	2	1.435 (0.255–8.065)	0.680
Malignancy	27	12	1.826 (0.849–3.924)	0.120
Chemotherapy	14	3	3.699 (1.020–13.421)	*0.035*
Invasive procedures				
Mechanical ventilation	67	38	1.824 (0.955–3.484)	0.067
Central catheterisation	74	54	0.872 (0.409–1.858)	0.723
Urinary catheterisation	94	66	1.424 (0.196–10.368)	0.726
Drugs used before diagnosis				
Penicillin and derivatives	29	15	1.529 (0.744–3.142)	0.246
3rd generation cephalosporins	32	23	1.067 (0.542–2.099)	0.852
Fluoroquinolones	22	5	3.646 (1.30–10.227)	*0.010*
Aminoglycosides	7	2	2.512 (0.504–12.510)	0.246
Carbapenems	49	37	0.840 (0.438–1.610)	0.599
Colistin	3	5	0.391 (0.090–1.699)	0.196

The value of *p* < 0.05 was set as the significance threshold (in italics)

*CI* confidence interval

### Antimicrobial susceptibility testing

The antimicrobial susceptibility test results of the 160 clinical and 12 environmental *A*. *baumannii* isolates were shown in Table 3. Four clinical isolates could not be regenerated during the study. A high proportion of the isolates were resistant to AN (91.8%), SAM (99.4%), CAZ (99.4%), CIP (100%) and IMP (99.4%) while most isolates were susceptible to COL (98.8%) and TIG (98.3%). Strains were considered as multidrug-resistant when the resistance was detected for at least one agent in three or more different categories of antimicrobials according to the definition of Magiorakos et al. [16].

**Table 3:** Antimicrobial susceptibility of invasive MDR *A. baumannii* isolates from ICUs (*n *= 172)

Antimicrobial agent	MIC_50_ (mg/L)	MIC_90_ (mg/L)	MIC range (mg/L)	Resistance (%)
Amikacin	≥ 256	≥ 256	1 to ≥ 256	91.8
Ampicillin–sulbactam	256	≥ 256	32 to ≥ 256	99.4
Ceftazidime	256	≥ 256	16 to ≥ 256	99.4
Ciprofloxacin	128	≥ 256	8 to ≥ 256	100
Imipenem	64	128	1 to ≥ 256	99.4
Colistin	0.5	1.0	0.25–64	1.2
Tigecycline	1.0	2.0	0.01–3	1.7

### Detection of antibiotic resistance genes

**Fig. 2:**
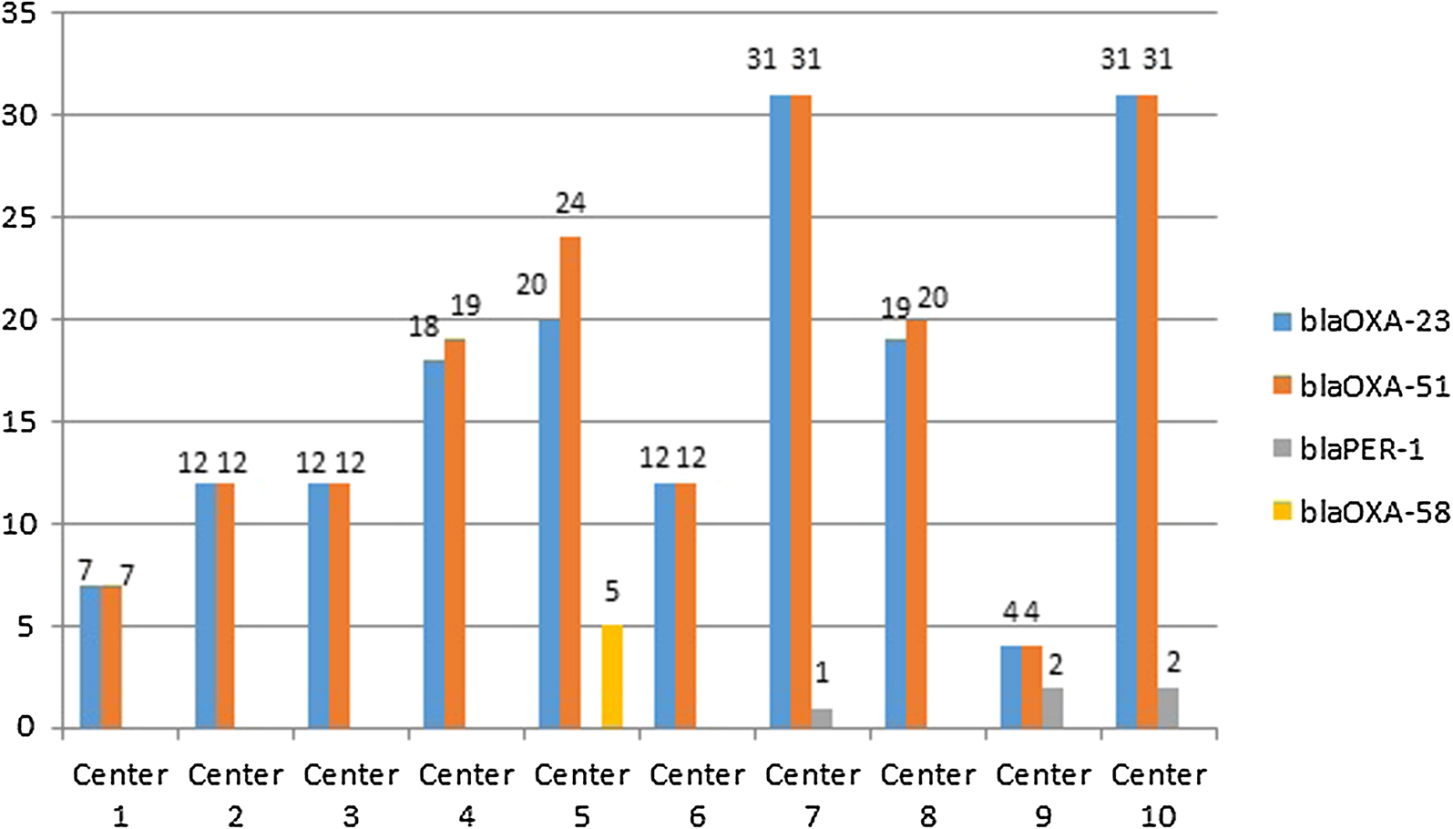
The frequency of resistance genes according to centers

All *A*. *baumannii* isolates possessed *bla*_OXA-51_ gene. The *bla*_OXA-23_ gene was detected in 166/172 (96%) isolates while *bla*_OXA-58_ was detected only in 5/172 (3%) isolates and none of the isolates had *bla*_OXA-24_ gene. One isolate carried both *bla*_OXA-23_ and *bla*_OXA-58_ genes. The *bla*_OXA-23_ was detected in all centers whereas *bla*_OXA-58_ gene was detected only in Center 5. The *bla*_PER-1_ gene was detected from three centers; Center 9 (*n *= 2), Center 10 (*n *= 2) and Center 7 (*n *= 1). None of the isolates showed *bla*_IMP_, *bla*_KPC_, *bla*_NDM_, and *bla*_OXA-48_ genes (Fig. 2).

### Molecular epidemiology

**Fig. 3:**
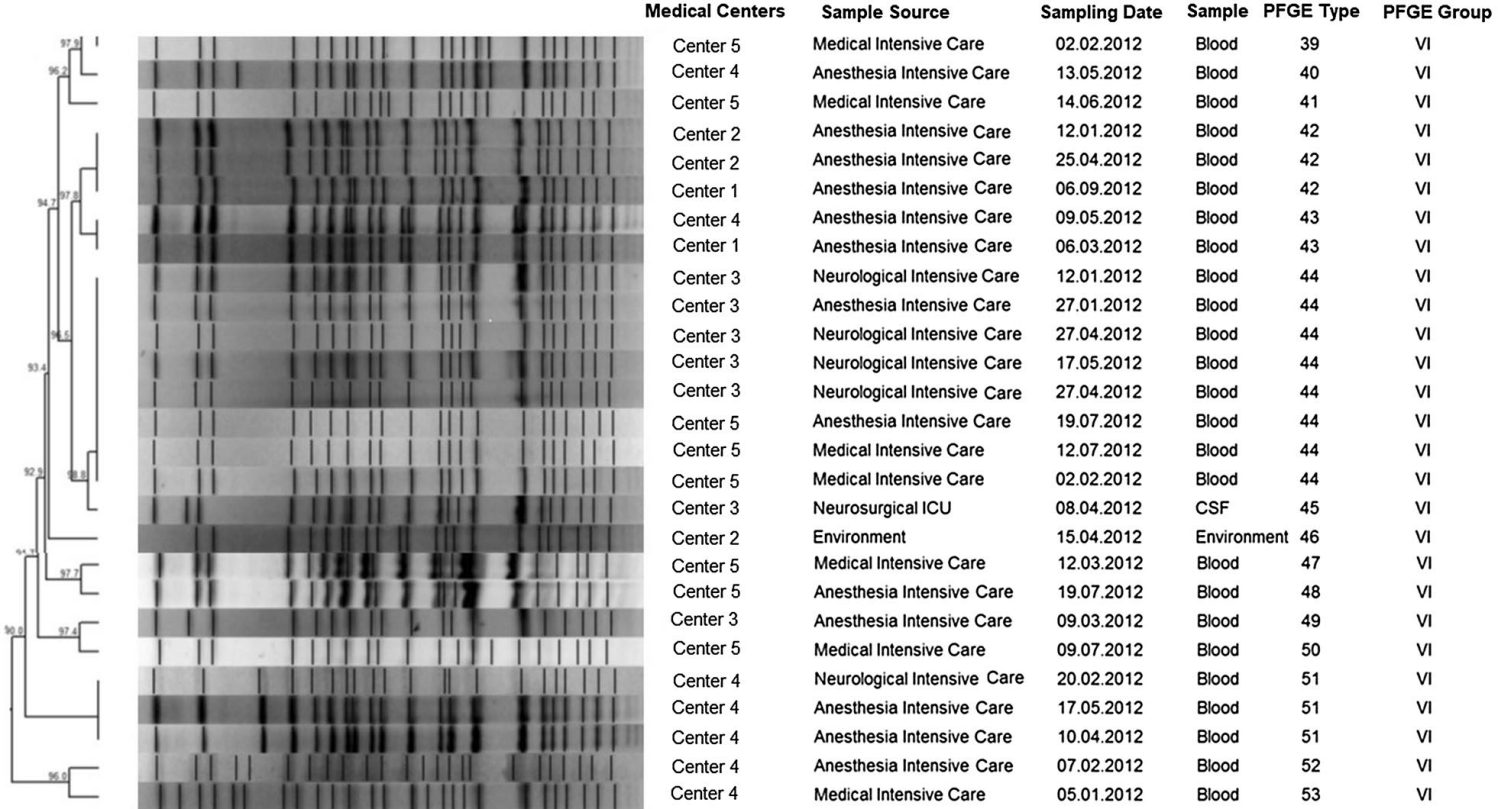
A cross-sectional example of PFGE dendogram representing the largest PFGE group (Group VI, *n *= 27). PFGE types; the strains having indistinguishable DNA band profile were clustered in the same PFGE types and labeled with a same number such as PFGE type 42 included 3 stains, any difference in PFGE patterns was defined as unique and labeled with different number. PFGE group VI included the strains having PFGE patterns that were similar to each other ≥ 85%

PFGE typing of the 172 isolates revealed 88 pulsotypes. Twenty-nine of these pulsotypes included two or more strains which could not be differentiated from each other. Totally 98 strains were classified in 29 pulsotypes, the clustering rate was 57%. When the similarity rate between the pulsotypes was assigned as ≥ 85%, 157 clonally related strains (91.2%) were clustered in 14 PFGE groups (I-XIV). Group VI (*n *= 27) was the largest among the PFGE groups followed by the groups II (*n *= 24) and IX (*n *= 21). Since dendrogram of all pulsotypes was very large, a cross-sectional example of dendrogram representing the most common PFGE group was shown as Fig. 3. Evaluation of the PFGE group types according to the centers revealed that group VI was dominant in three centers (Center 3, Center 4, and Center 5) and was the second most dominant type in two centers (Center 1 and Center 2). The clinical (*n *= 10) and environmental (*n *= 2) isolates recovered from different ICUs of Center 6 were in the PFGE group IV and these isolates were all identical. In five centers located in Central Anatolia, group VI was the dominant group. Group II was detected more often in two centers (Center 9 and Center 10). In group XII, isolates were only from Center 7, and 11 of these isolates were obtained from the burns unit. Center 8 differed from the other centers since the dominant group type was XIV for this center.

## Discussion

*Acinetobacter baumannii* has become one of the most serious threats especially in the ICUs of the hospitals being respondless to antibiotic therapy. Therefore, new strategies should be adopted to combat this pathogen. At this point, data obtained from especially multicenter studies is needed to understand the epidemiology of *A*. *baumannii*. The examination of demographic characteristics of patients with MDR *A*. *baumannii* infections, detection of resistance genes and the spread of resistant strains in hospitals can help to control these infection. In this study, epidemiological characteristics of invasive *A*. *baumannii* strains from 10 medical centers in five different geographical regions of Turkey were evaluated. To our knowledge, the current study is the most comprehensive epidemiological study in Turkey in terms of distribution and number of participant centers and isolates.

The important risk factors for mortality in patients with *A*. *baumannii* infections were long-term medication in ICUs, APACHE II score > 20, ventilator associated bacteremia, use of third generation cephalosporins before the diagnosis of infection and liver cirrhosis [17]. In our study, being > 65 age, having DM or CLF, taking chemotherapeutic drugs or fluoroquinolones before diagnosis were found to be related with mortality. On the contrary, using carbapenem or third generation cephalosporins was not found to be related to mortality in the studied patient population.

According to surveillance studies, carbapenem resistance is a major problem in the treatment of *A*. *baumannii* infections especially in Turkey, Greece, Italy, Spain, Romania and the UK [2, 18]. In our study, colistin was the most effective antimicrobial agent against MDR *A. baumannii* isolates, confirming the multicenter study which SENTRY performed between 2001 and 2011 and it was reported that colistin had low level resistance (0.9–3.3%) and was still effective against MDR *A*. *baumannii* isolates [19].

Among the resistance genes responsible for carbapenem resistance in *A*. *baumannii* isolates, *bla*_OXA-23_, *bla*_OXA-24_ and *bla*_OXA-58_ are the top leading ones. The dominance of these genes in resistant *A*. *baumannii* isolates has been changed all over the world. The predominance of *bla*_OXA-58_ between 1999 and 2009 in Mediterranean countries like Italy, Greece, Lebanon and Turkey has showed a transition to *bla*_OXA-23_ since 2009 causing an increase in the frequency of *bla*_OXA-23_. This rise caused of a higher carbapenem MIC_50_ and MIC_90_ values between 2010 (16 mg/L and 256 mg/L, respectively) and 2011 (128 mg/L and 256 mg/L) [20]. There are some reports about the rise of *bla*_OXA-23_ gene in carbapenem resistant strains of *A*. *baumannii* in Turkey [21, 22]. Although the study populations and duration time of these studies had some differences, there was a consensus about the increasing number of MDR *A*. *baumannii* isolates which have *bla*_OXA-23_ gene. As the *bla*_OXA-23_ enzyme was found in all centers but *bla*_OXA-58_ only in one center, the results of the current study highlighted that *bla*_OXA-23_ gene is the most prevalent oxacillinase in Turkey.

The frequency of *bla*_PER-1_ can vary from country to country. In Turkey according to the local studies, the frequency of *bla*_PER-1_ among MDR *A*. *baumannii* isolates has been in a declining trend (52.9% to 8.3%) since 2008. In our study, the frequency of *bla*_PER-1_ was found to be 2.8%. According to previous reports, the annual rate of PER-1 detection decreased gradually over time and the most prominent decrease occurred in 2010 in Turkey. The frequency of PER-1 type β-lactamases in *A. baumannii* species has decreased, and PER-1 is no longer a threat in Turkey’s resistance profile [23].

The important feature in the surveillance of MDR *A*. *baumannii* isolated from patients with hospital infections is the typing of strains by using a standard method such as PFGE. The use of PFGE especially in multicenter studies is very important to estimate the clonal relatedness between isolates obtained from different centers. A previous study revealed that 60‒80% of the strains were clonally related throughout the country [24]. In the current study, the PFGE results of the clinical and environmental MDR *A*. *baumannii* strains isolated from the ICUs of Center 6, Center 7 and Center 10 revealed the same pulsotype although the regions of these centers are far away from each other. Furthermore, one of the clinical isolates of Center 10 was identical to the environmental isolate of Center 2, which is quite interesting since the regions are also far away from each other.

Some of the strains which were responsible for mini epidemics in ICUs of different centers showed the same pulsotype profile with 91.2% relatedness and this result revealed that these isolates did not differ so much from each other. Detection of the same or very similar pulsotypes from different centers suggests that infection control measures should be strengthened and revised in our country. This situation can be achieved with the measures indicated in the report of Cheon et al. which described the control of endemic MDR *A*. *baumannii* outbreaks in ICUs within 1 year with comprehensive and intensive infection control measures such as effective antibiotic management, personnel training, contact precautions, hand hygiene and active surveillance cultures of hospital environmental samples [25].

The current study also has some limitations. Although ten centers from five different regions of Turkey were included in the study, further studies with the participation of more centers are needed. Moreover, the mortality rates in the current study might be different to those in other comprehensive studies since almost all patients were immunosuppressive and had underlying diseases.

In conclusion, the results of this study indicates that clinical and environmental *A*. *baumannii* isolates obtained from different centers in Turkey are closely related to each other, the resistance against carbapenems is by far the most important emergency for *A. baumannii* infections and *bla*_OXA-23_ has become the most prevalent carbapenemase in Turkey.

## Data Availability

Not applicable.
